# Genotypic and Phenotypic Characteristics of Lactic Acid Bacteria Associated with Forage Plants in the Native Grassland of Western Inner Mongolia and Their Application for Alfalfa Silage Fermentation

**DOI:** 10.3390/ani14101394

**Published:** 2024-05-07

**Authors:** Wenlong Li, Feng Li, Chen Zhang, Jie Gao, Ya Tao

**Affiliations:** 1 Institute of Grassland Research, Chinese Academy of Agricultural Sciences, Hohhot 010010, China; liwenlong@caas.cn (W.L.); lifeng03@caas.cn (F.L.); zhangchen05@caas.cn (C.Z.); gaojie03@caas.cn (J.G.); 2 Key Laboratory for Model Innovation in Forage Production Efficiency, Ministry of Agriculture and Rural Affairs, Hohhot 010010, China; 3 National Center of Technology Innovation for Dairy, Hohhot 010090, China

**Keywords:** Inner Mongolia, natural forage plant, *Lactiplantibacillus plantarum*, *Pediococcus pentosaceus*, alfalfa silage

## Abstract

**Simple Summary:**

Western Inner Mongolia is a principal part of the arid region in northwest China, covered mainly with semifrutex and frutex plants in the native grassland. Owing to the fact that epiphytic lactic bacteria (LAB) species are sensitive to the environment, the low nutrient concentrations, low water availability, and large changes in temperature around the phyllosphere in this region may lead to LAB species with special properties. However, limited information is available on the biodiversity and ensiling parameters of LAB on the forage plants in this region. This study was conducted to investigate the genotypic and phenotypic characteristics of LAB associated with forage plants in the region, and to screen for efficient strains for well-preserved alfalfa silage. A total of 73 strains belonging to 16 species were isolated, and most of strains could grow at 5–45 °C and in 6.0% NaCl, possessed good cryotolerance and osmotolerance. *Lactiplantibacillus plantarum* subsp. *plantarum* (GI19) and sucrose was the most effective combination for alfalfa ensiling, with a higher lactic acid content and lower pH, undesirable microorganism counts, and acetic acid and NH_3_-N contents. This additive could be used to produce quality alfalfa silage for the region to alleviate the shortage of feed in autumn and winter.

**Abstract:**

This study was conducted to investigate the genotypic and phenotypic characteristics of lactic acid bacteria (LAB) associated with forage plants in the native grassland of western Inner Mongolia and to evaluate their effects on alfalfa silage fermentation. Forage plants and their spontaneous fermentation silages were analysed using culture-based techniques for LAB isolation; the phenotypic properties and 16S rDNA and *phe*S or *rpo*A gene sequences of the isolates were evaluated; alfalfa was ensiled with four additive combinations: *Lactiplantibacillus plantarum* subsp. *plantarum* (GI19), *Lact. plantarum* subsp. *plantarum* and *Pediococcus pentosaceus* (GI19+GI51), GI19 and 20 g/kg fresh matter of sucrose (GI19+S), and GI19+GI51+S, for 60 d. A total of 73 strains belonging to 16 species were isolated. All isolates grew at 5–45 °C and in 3.0% NaCl, and most of them grew in 6.5% NaCl. *Enterococcus faecalis* and *Lact. plantarum* were 26.03% and 17.81% of the total isolates, respectively. All additives improved the silage quality, while GI19+S was more effective for alfalfa ensiling with a higher lactic acid content and lower pH, undesirable microorganism counts, and acetic acid and NH_3_-N contents than remnant additives. In conclusion, the LAB species were diverse, and most of them possessed good cryotolerance and osmotolerance; GI19+S was the optimal inoculant for alfalfa fermentation improvement.

## 1. Introduction

Silage is a common source of livestock feed globally, and it is driven by lactic acid bacteria (LAB), which produce adequate amounts of acid, decrease pH, and inhibit the growth of undesirable microorganisms [1]. Because the main forage plant types in this area, such as Leguminosae, Chenopodiaceae, and Asteraceae, contain relatively low sugar contents, successful spontaneous ensiling for some forages in this region is difficult. For enhancing silage preservation, available exogenous LAB were collected, characterised, and applied from LAB-related resources for ensiling, which are attracting increasing attention in silage research [2].

LAB in forage and silage display extremely abundant biodiversity, and several new species have been recently isolated from silage; some of these show a high performance for producing well-preserved silage [3]. Epiphytic LAB species vary among standing plants; they are affected by the chemical composition and morphology of the host plant [4,5] and are sensitive to the environment. Therefore, extensive collection, identification, characterisation, and screening of LAB from different plants and environments provides critical strains for efficient LAB or probiotic inoculants, which is of great significance to improving the silage quality and promoting livestock health. Western Inner Mongolia is a principal part of the arid region in northwest China. Owing to its low precipitation and unique climatic conditions, it forms a distinct ecological region covered principally with semifrutex and frutex plants in the native grassland. Although the low nutrient concentrations, low water availability, and large changes in temperature around the phyllosphere are challenging environments for the epiphytic LAB in this region [6], these harsh conditions may lead to the selection of LAB species with special properties. However, limited information is available on the biodiversity and ensiling parameters of LAB on the forage plants in this region. Moreover, LAB autochthonous to the plant environment are better suited for plant-based fermentation compared to allochthonous strains [7]. In western Inner Mongolia, LAB encounter environmental stresses on natural forage plants and in their spontaneous fermentation silages, and may have more effects on improving the fermentation of local forage plants or forage with a lower sugar content. Therefore, it is necessary to study the presence and characteristics of LAB in this ecological district associated with forage plants, and to screen efficient strains for producing well-preserved silage.

As a primary forage produced in western Inner Mongolia, high-quality silage of alfalfa (*Medicago sativa* L.) is crucial for local livestock production. However, alfalfa is challenging to ensile, owing to its relatively low abundance of epiphytic LAB and water-soluble carbohydrate (WSC) content. The additions of LAB and sugar are fundamental methods to solve these problems. Silages treated with one or more of the facultative heterofermentative LAB always show good fermentation quality, and they may show potential synergistic effects with multiple inoculants [8]. Thus, screening of the LAB associated with the local native forage plants could be a feasible strategy to improve the ensiling quality of alfalfa in the region. Moreover, sucrose combined with LAB has been suggested to be more efficient with regard to legume ensiling than a LAB inoculant alone for directly supplying sufficient substrates to the growth of LAB [9,10].

The present study aimed to investigate the taxonomic status and characteristics of LAB associated with forage plants in the native grassland of western Inner Mongolia, and to evaluate the effects of LAB, single or multiple, on improving alfalfa silage quality with or without sucrose addition.

## 2. Materials and Methods

### 2.1. Samples and LAB Isolation

**Figure 1:**
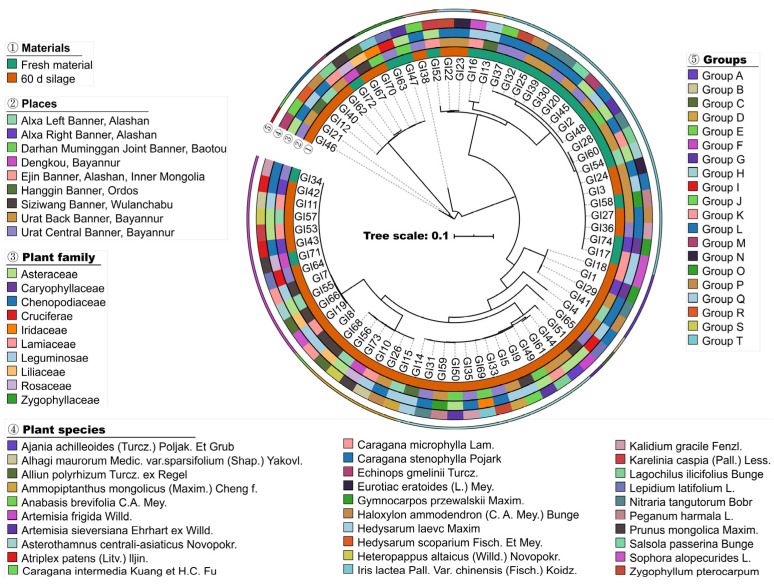
Phylogenetic development tree map of 73 lactic acid bacteria strains isolated from forage plants grown in the native grassland of western Inner Mongolia and their spontaneous fermentation silages. Four different inner circles (①–④) show the sources of LAB, including materials, the collected places of samples, and the family and species of the forage plants. The groups of the isolates are labelled in the outer circle (⑤).

Thirty-seven natural forage plant samples belonging to 10 families or 30 species were obtained from 9 counties of the native grassland in western Inner Mongolia, China (Figure 1, Table S1). Hetero-plants were removed from each sample to ensure pure species in each sample. The samples were then ensiled, respectively, as follows: the forage plants were chopped into about 2 cm pieces, mixed thoroughly, sub-sampled with three replicates, and directly frozen at −20 °C for later examination. Approximately 300 g of chopped materials were packed into plastic film bags (250 × 360 mm, Shijiazhuang Xilong Packing Co., Ltd., Shijiazhuang, China) and sealed with a vacuum sealer (DZ-260, Beijing Jod Packing Machinery Co., Ltd., Beijing, China). Three bags were made per natural forage plant sample, and all were stored at ambient temperature (20–25 °C) for 60 d.

LAB were isolated by dilution and plating, using the method described by Cai et al. [11], from fresh forage plants and their silages. Representative colonies of all morphologies were taken randomly and purified twice by streaking on de Man, Rogosa, Sharpe (MRS) agar (Difco Laboratories, Detroit, MI, USA), then resuspended in nutrient broth (Difco) with dimethyl sulfoxide at a ratio of 9:1, and stored as stock cultures at −80 °C for further analysis.

### 2.2. Phylogenetic Relationships among Isolates

The isolated LAB were cultivated overnight in MRS agar for DNA extraction. The complete genomic DNA of each of the LAB strains was extracted using a TIANamp Bacterial DNA kit (Tiangen Biotech, Beijing, China) according to the instructions. The partial 16S rDNA was amplified with primers 27f (5′-AGA CTT TGA TCC TGG CTC AG-3′) and 1492r (5′-TAC GGC TAC CTT GTT ACG ACT-3′), and 2×Taq PCR MasterMix (Tiangen) using polymerase chain reaction (PCR) in a thermal cycler (Veriti 96-Well Thermal Cycler, Thermo Fisher Scientific, Singapore) [12]. The amplification of *phe*S and *rpo*A genes was carried out as indicated by Naser et al. [13], using the primers rpoA-21-F/rpoA-23-R (5′-CAYCCNGCHCGYGAYATGC-3′/5′-GGRTGRACCATVCCNGCHCC-3′) and *phe*S-21-F/*phe*S-23-R (5′-ATGATYGARTTTGAAAAACC-3′/5′-ACHGTRTTRATDCCDGCRCG-3′). The PCR product sequences were analysed by Personalbio Co., Ltd. (Shanghai, China). The data from the 16S rDNA sequences of the isolates in this study were deposited in GenBank with the accession numbers MT158579 to MT158651.

The 16S rDNA sequences of all LAB isolates were aligned using Multiple Sequence Alignment Software Version 7 [14], and a maximum likelihood phylogenetic tree was constructed using RAxML v8.2.9 with 1000 bootstraps [15]. Interactive Tree of Life (iTOL) was used for displaying and annotating the phylogenetic trees [16]. Four different circles outside of the tree illustrated the sources of LAB, including materials, the collected places of samples, and the family and species of the forage plants, annotated in different colours.

### 2.3. Morphological, Physiological, and Biochemical Tests of LAB

For morphological, physiological, and biochemical tests, LAB were activated after 24 h of incubation in MRS agar. Gram strain, catalase activity (3% H_2_O_2_), LAB morphology, and gas production from glucose were performed as described by Kozaki et al. [17]. The growth of LAB in MRS broth (Difco) at temperatures 5 °C and 10 °C was observed after incubation for 14 d, and at 40 °C and 45 °C for 7 d. Growth of LAB at pH values of 3.0, 3.5, 4.0, 4.5, 5.0, 7.5, and 8.0 was observed in MRS broth, in which pH was adjusted using 1 mol/L HCl or NaOH, after incubation at 30 °C for 7 d. Salt tolerance was observed in the MRS broth containing 3.0% and 6.5% of NaCl at 30 °C for 2 d. Nontreated cultures were used as controls for aforesaid assays, and whether there had been growth, no growth, or weak growth was determined. Carbohydrate fermentation tests were carried out using API 50 CH strips (BioMerieux, Tokyo, Japan) according to the manufacturer’s instructions, and the results were observed after incubation at 30 °C for 48 h. For assessing the potential fermentation parameters of the isolates, acidification was investigated in MRS broth inoculated with 2% (*v*/*v*) isolate cultures, and pH was measured after 12, 24, and 48 h of incubation at 30 °C using a pH meter (UB-7, Denver Instruments, Denver, CO, USA).

### 2.4. Species Identification by 16S rDNA Sequencing, pheS and rpoA Genes

The resulting sequences of 16S rDNA and *phe*S and *rpo*A genes were compared with the LAB type strains from GenBank using the Basic Local Alignment Search Tool (BLAST). Nucleotide substitution rates (Knuc values) were calculated, and phylogenetic trees were constructed using the neighbour-joining distance method, whose bootstrap analysis of sequence data was based on 1000 random resamples using Molecular Evolutionary Genetics Analysis (MEGA) 5.0 software (The Biodesign Institute, Tempe, AZ, USA).

### 2.5. Silage Preparation and Microbial and Chemical Analysis

To evaluate fermentation capacity, overnight cultures of the strains were inoculated in alfalfa broth at 30 °C for 24 h. Cultures were taken to record the growth rate, pH value, and contents of organic acid as described by Li et al. [18]. The strains GI19 and GI51 were selected as inoculants due to their higher lactic acid production activity and growth rate in alfalfa broth than the remaining isolates. For alfalfa silage preparation, the alfalfa was harvested at the early bloom stage (10% flowering rate) from a farm in Bayannur, Inner Mongolia, and the additives were designed as follows: 1.0 × 10^6^ cfu/g fresh matter (FM) of GI19, 1.0 × 10^6^ cfu/g FM of GI19 and GI51, respectively (GI19+GI51), 1.0 × 10^6^ cfu/g FM of GI19 and 20 g/kg FM of sucrose (GI19+S), and 1.0 × 10^6^ cfu/g FM of GI19 and GI51 and 20 g/kg FM of sucrose (GI19+GI51+S). Additives were dissolved and sprayed on chopped alfalfa, and a control was added with the same volume of distilled water. 

After 60 days of ensiling at ambient temperature (20–25 °C), a 20 g sample from each bag silo was blended with 180 mL of sterilized NaCl solution (0.85% *w*/*v*), serial decimal dilutions were obtained, and the microbial populations of the fresh materials and silages were measured by the plate count method on lactobacilli de Man, Rogosa, Sharpe (MRS) agar (Difco Laboratories, Detroit, MI, USA), blue light agar (Nissui-seiyaku, Tokyo, Japan), potato dextrose agar (Nissui-seiyaku), and nutrient agar (Nissui-seiyaku) according to Yang et al. [19]. The pH levels of the filtrates were measured using a pH meter (UB-7, Denver Instruments, Denver, CO, USA), and the contents of organic acid (lactic acid, acetic acid, propionic acid, and butyric acid) were analysed by high-performance liquid chromatography (HPLC; LC-10A, Shimadzu, Co., Ltd., Kyoto, Japan; column: Shodex RS pak KC-811, Showa Denko K.K., Tokyo, Japan; detector: DAD, 210 nm, SPD-20A; eluent: 3 mmol/L HClO_4_, 1.0 mL/min; temperature: 50 °C). A 10 mL aliquot of 250 g/L (*w*/*v*) trichloroacetic acid (TCA) was added to 40 mL of fermentation filtrate and mixed well. The mixture was left to stand at ambient temperature (25 °C) for 1 h to precipitate protein and then centrifuged at 18,000× *g* for 15 min at 4 °C. The supernatant fluid was analysed for ammonia-N and free amino acid N (FAAN) according to the ninhydrine assay of Broderick and Kang [20], with leucine as amino acid standard. The peptide-N content was determined by the increase in FAA in the TCA supernatant after digesting with 6 mol/L hydrochloric acid for 21 h at 105 °C under an N_2_ atmosphere [21]. The concentration of NPN was quantified by measuring the amount of true protein precipitated by TCA according to the method of Licitra, Hernandez, and Soest [22]. Dry matter (DM) was determined by oven drying at 65 °C for 48 h. In addition, the chemical compositions of crude protein (CP) [23], WSC [24], neutral detergent fibre (NDF), and acid detergent fibre (ADF) [25] were also measured.

### 2.6. Statistical Analysis

Cluster analysis was conducted based on the isolate parameters of cold, hot, salt and acid tolerance, and API 50 CH fermentation patterns using the complete linkage method, and aimed to test the similarities and differences in phenotypic characteristics between representative strains of 20 groups isolated from forage plants grown in native grassland of western Inner Mongolia and their spontaneous fermentation silages. Results were further visualised as a heatmap using the imageGP, a free online platform for data analysis http://www.ehbio.com/ImageGP (accessed on 28 November 2023). The effects of LAB inoculation and sucrose addition on the microbial populations, chemical compositions, fermentation characteristics, and NPN fractions were tested using two-way analysis of variance with the fixed main effects of sucrose, LAB, and their interaction using the general linear model of IBM SPSS Statistics (SPSS 24.0, SPSS Inc., Chicago, IL, USA). Tukey’s honest significance test was conducted to compare the differences between means. The effects were considered statistically significant at *p* < 0.05.

## 3. Results

### 3.1. Isolation of LAB

Thirty forage species were collected from nine counties (Figure 1, Table S1), for a total of 37 samples. Leguminosae, Chenopodiaceae, and Asteraceae showed higher specie abundance than that of other families in the samples, being 26.7%, 26.7%, and 23.3%, respectively. Based on gram-positive, catalase-negative, and morphological tests, 73 strains isolated from forage plants grown in the native grassland of western Inner Mongolia and their spontaneous fermentation silages were selected from representative colonies as candidate LAB. They could be isolated from silages of all species except for *Salsola passerina* Bunge. Moreover, LAB were not detected in parts of the fresh materials, including five out of eight forage specie samples in the Leguminosae, two out of eight in the Chenopodiaceae, and three out of seven in the Asteraceae, as well as *Alliun polyrhizum* Turcz. ex Regel and *Iris lactea Pall.* var. *chinensis* (Fisch.) Koidz. In addition, no LAB were isolated from the fresh forage samples grown in Siziwang Banner.

### 3.2. The Morphological, Physiological, and Biochemical Properties of LAB Strains

According to the similarity in their 16S rDNA gene sequences, as well as the physiological and biochemical properties of the LAB isolates, all the strains were clustered into 20 groups (Figure 1). The representative strains of each group were selected based on phenotypic characteristics (Table 1). Groups A to F were rods, and strains in groups A, B, C, E, and F were homofermentative strains that did not produce gas from glucose. The pH in their MRS broth decreased to below 4.2 within 12 h and lowered below pH 4.0 after 24 h incubation (except group A). In contrast, the strains in group D were heterofermentative, and the pH decreased at 4.8 in MRS broth within 48 h incubation, making them different from other groups. All cocci-shaped isolates were distributed in groups G to T. The strains in groups G, H, P, Q, R, S, and T showed similar gas production from glucose as homofermentative cocci, whereas groups I-O were heterofermentative. The pH of MRS broth, inoculated with the GI51 (group G), dropped to 4.1 and 3.9 after 12 h and 48 h fermentation, respectively.

**Figure 2:**
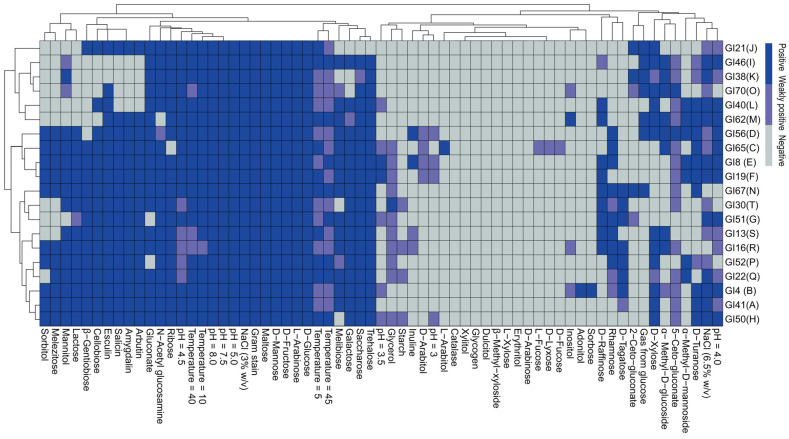
Clustering of the phenotypic characteristics of the representative strains in each of the groups isolated from forage plants grown in the native grassland of western Inner Mongolia and their spontaneous fermentation silages. The dendrogram was constructed based on physiological and biochemical parameters and the API 50 CH fermentation patterns of lactic acid bacteria strains. The colour scale showed positive with blue, negative with azure, and weakly positive with slateblue.

A heatmap was applied to visualise the physiological and biochemical properties of the representative LAB strains (Figure 2) and to cluster the LAB strains into two primary clusters. The first cluster was composed of groups I, J, K, L, M, and O, which could not metabolize lactose, while all the other groups belonged to the second cluster, which divided into two further sub-clusters. The first sub-cluster was composed of groups C, D, E, and F, which could metabolize D-arabitol weakly. Moreover, the phenotypic characteristics and their relationships are presented on the horizontal axis of the heatmap. All isolates were able to grow at 5 °C to 45 °C. The rod-shaped isolates were found to be capable of growing in 6.5% NaCl and pH 4.0 to 8.0, but could not grow or grew weakly at pH 3.0. The cocci-shaped isolates were able to grow in 3.0% NaCl, and at pH ranging from 4.5 to 8.0, while the strains in group G, H, I, L, and M were all able to grow well at pH 4.0, and only the strains in group R and N could not grow in 6.5% NaCl. All isolated strains were positive for L-arabinose, D-glucose, D-fructose, D-mannose, and maltose, but negative for erythritol, D-arabinose, L-xylose, β-methyl-xyloside, dulcitol, glycogen, and xylitol.

### 3.3. Phylogenetic Analysis of 16S rDNA Sequence

**Figure 3:**
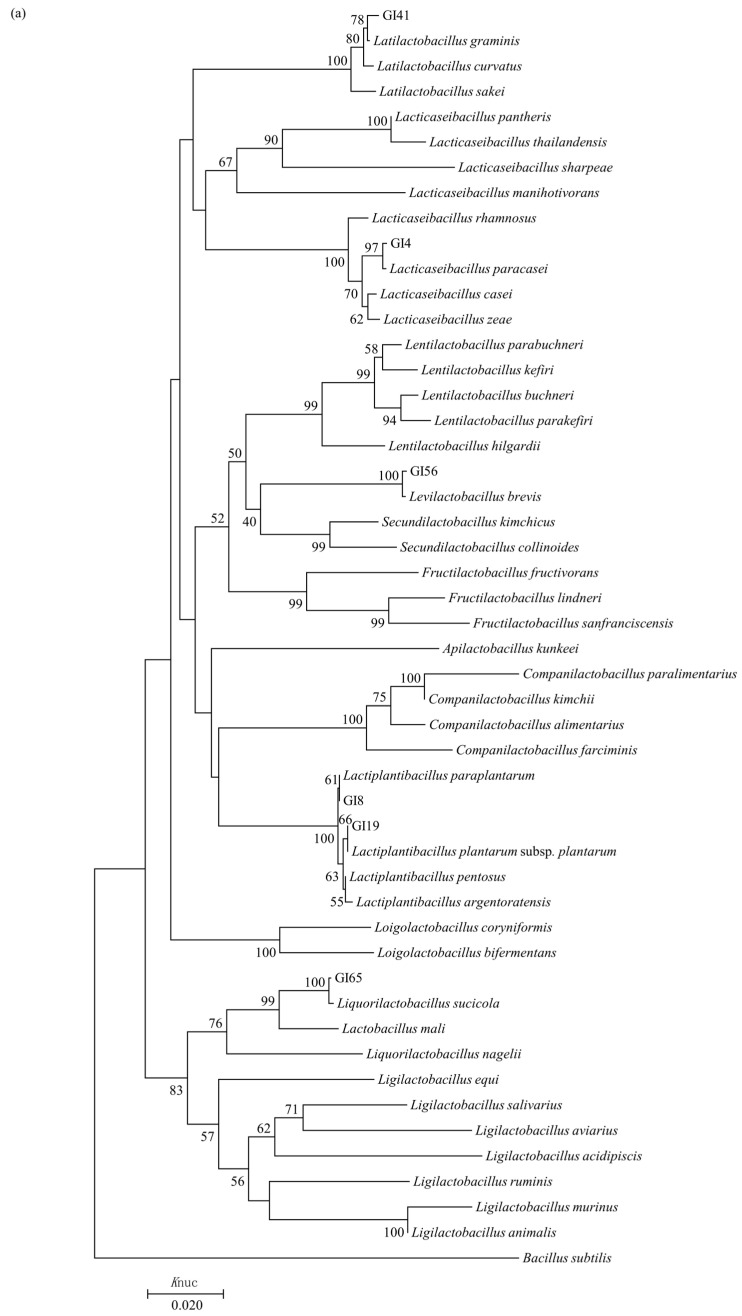

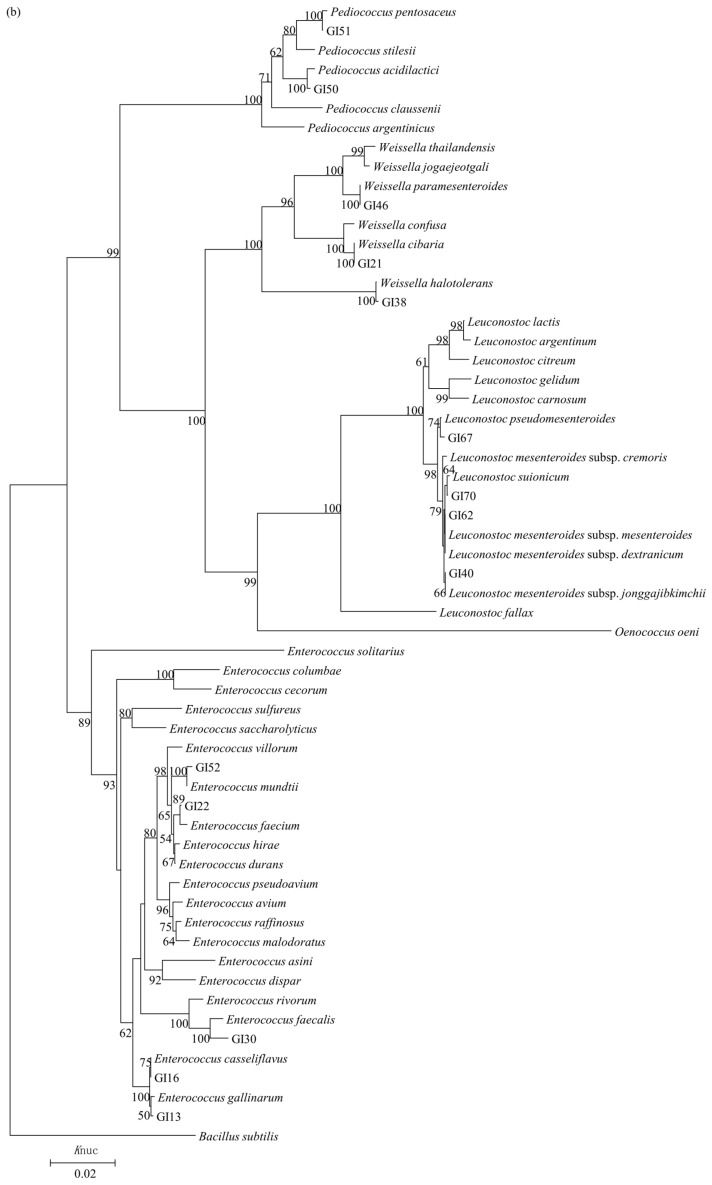
(**a**) Phylogenetic tree showing the relative position of representative rod-shaped strains isolated from forage plants grown in the native grassland of western Inner Mongolia and their spontaneous fermentation silages. (**b**) Phylogenetic tree showing the relative positions of representative cocci-shaped strains isolated from forage plants grown in the native grassland of western Inner Mongolia and their spontaneous fermentation silages. Neighbour-joining method was used with 16S rDNA sequences. Bootstrap values for a total of 1000 replicates are shown at the nodes of tree. *Bacillus subtilis* was used as an out-group. The bar indicates 1% sequence divergence; *K*nuc, nucleotide substitution rates.

Following molecular phylogeny analysis of the representative strains in each group, all rod and cocci-shaped isolates could be grouped into five different genera, including *Lactobacillus*, *Eenterococcus*, *Pediococcus*, *Weissella*, and *Leuconostoc* (Figure 3). Strain GI41 in group A formed a distinct cluster together with *Latilactobacillus graminis* type strains, supported by 78.00% bootstrap values (Figure 3a). Group B (strain GI4) was placed in the cluster together with *Lacticaseibacillus paracasei* with 97.00% bootstrap support. Strain GI65 and GI56, representative of group C and D, formed a well-defined cluster with *Liquorilactobacillus sucicola* and *Levilactobacillus brevis* type strains, respectively, in a 100.00% bootstrap cluster on the phylogenetic tree. Strains GI8 and GI19 in groups E and F were categorised in the *Lact. plantarum* cluster, grouped on the phylogenetic tree together with *Lact. paraplantarum*, *Lact. plantarum* subsp. *plantarum*, *Lact. Pentosus*, and *Lact. plantarum* subsp. *argentoratensis* with similarity greater than 99.65%.

Strains GI51 and GI50 from groups G and H fell in the cluster of the genus *Pediococcus*, and were identified as *Ped. pentosaceus* and *Ped. acidilactici* with 100.00% and 99.00% bootstrap support, respectively (Figure 3b). The strains GI46, GI21, and GI38 in group I, J, and K were placed in the genus *Weissella*, and were phylogenetically closest to the species *W. paramesenteroides*, *W. cibaria*, and *W. halotolerans*, respectively, all of which were supported by over 100.00% bootstrap values. Groups L, M, N, and O were characterised as the genus *Leuconostoc*, and strain GI67 from group N was placed in the cluster together with *Leuc. pseudomesenteroides* in a 74.00% bootstrap cluster. Strain GI40 and GI70 in groups L and O formed a distinct cluster together with *Leuc. mesenteroides* subsp. *Jonggajibkimchii* and *Leuc. mesenteroides* subsp. *suionicum* showed 100.00% and 99.93% similarities, respectively. Group M (strain GI62) grouped on the phylogenetic tree together with *Leuc. mesenteroides* subsp. *dextranicum* and *Leuc. mesenteroides* subsp. *mesenteroides*, which presented 99.86% to 100.00% similarity. Groups P, Q, R, S, and T were placed in the *Enterococcus* cluster in the phylogenetic tree. The strains GI52, GI22, GI16, and GI30, representative of groups P, Q, R, and T, showed *Ent. mundtii*, *Ent. faecium*, *Ent. casseliflavus*, and *Ent. faecalis* as the most closely related species, supported with bootstrap support greater than 75.00%. Strain GI13 in group S was grouped on the phylogenetic tree together with *Ent. gallinarum* and *Ent. casseliflavus*, which showed 99.93% similarity.

### 3.4. Phylogenetic Analysis of pheS and rpoA Genes

**Figure 4:**
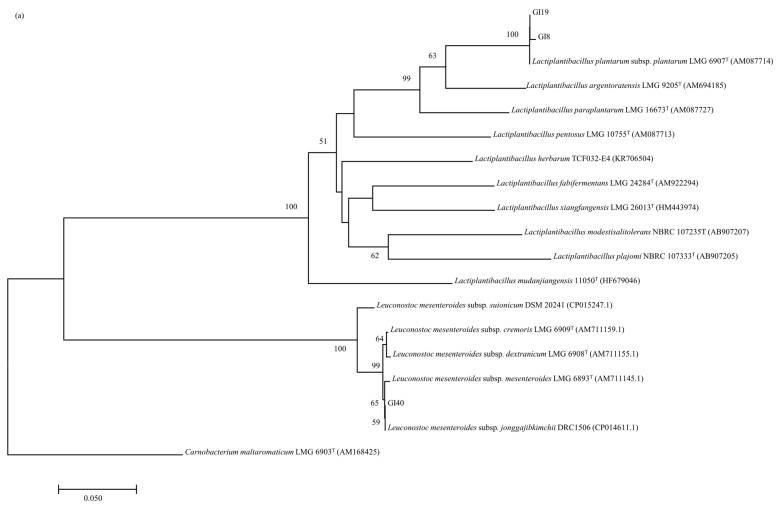

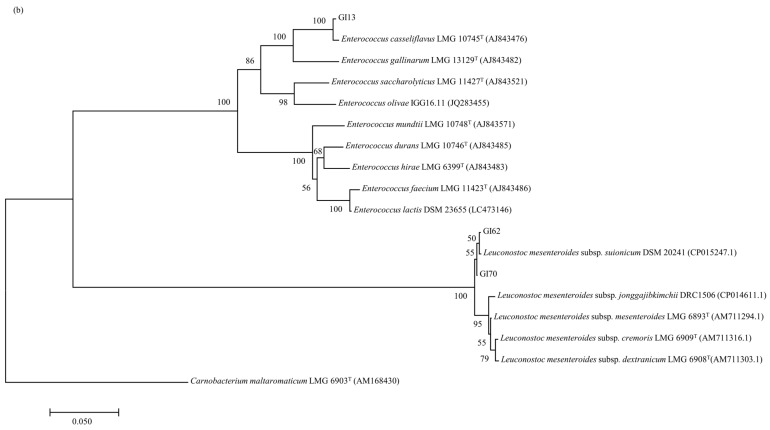
(**a**) Phylogenetic trees based on partial *pheS* gene sequences of representative strains isolated from forage plants grown in native grassland of western Inner Mongolia and their spontaneous fermentation silages. (**b**) Phylogenetic trees based on partial *rpoA* gene sequences of representative strains isolated from forage plants grown in native grassland of western Inner Mongolia and their spontaneous fermentation silages. Neighbour-joining method was used with *pheS* or *rpoA* gene sequences. Bootstrap values for a total of 1000 replicates are shown at the nodes of tree. *Carnobacterium maltaromaticum* was used as an out-group. The bar indicates 5% sequence divergence; *K*nuc, nucleotide substitution rates.

According to the results of the *phe*S gene analysis (Figure 4a), the strains GI8 and GI19 in groups E and F were placed in the cluster together with *Lact. plantarum* subsp. *plantarum* and *Lact. plantarum* subsp. *argentoratensis*. Since GI8 presented 99.00% and 89.72% similarity with respect to these type strains, and GI19 showed 98.74% and 89.65% similarity with *Lact. plantarum* subsp. *plantarum* and *Lact. plantarum* subsp. *argentoratensis*, respectively, GI8 and GI19 were all identified as *Lact. plantarum* subsp. *plantarum* with more than 10.00% genetic difference from *Lact. plantarum* subsp. *argentoratensis*. The strain GI40 from group L presented 99.20% similarity with respect to the type strain of *Leuc. mesenteroides* subsp. *Jonggajibkimchii*, and values ranging from 98.93% to 98.66% with respect to the type strains of *Leu. mesenteroides* subsp. *cremoris*, *Leuc. mesenteroides* subsp. *mesenteroides*, and *Leuc. mesenteroides* subsp. *dextranicum*, so it was assigned to *Leuc. mesenteroides* subsp. *Jonggajibkimchii*.

The phylogenetic analysis of the *rpoA* genes is shown in Figure 4b. Strain GI13 from group S was more closely related to the type strain of *Ent. casseliflavus*, with 99.41% similarity, than to the type strains of *Ent. gallinarum*, with a similarity value of 93.80%, and was assigned to *Ent. ca*sseliflavus. The strains GI62 and GI70, representative of groups M and O, were all identified as *Leuconostoc mesenteroides* subsp. *suionicum* with 99.74% similarity, having values ranging from 97.77% to 98.29% with respect to the type strains of the remaining subspecies.

### 3.5. Fermentation Characteristics of Alfalfa Silages Prepared with Additives

For fresh alfalfa material, the contents of DM, CP, WSC, and NPN were 25.65%, 17.87%, and 5.82% on a DM basis, and 26.34% of the TN content. The count of LAB was 3.30 log_10_ cfu/g FM, while the counts of aerobic bacteria, coliform bacteria, yeast, and mold were 5.10, 4.62, 3.25 and 3.00 log_10_ cfu/g FM, respectively (Table 2). After 60 d of ensiling, the silages inoculated with GI19 and GI19+GI51 showed decreased pH, contents of propionic acid, butyric acid, and NH_3_-N and increased contents of lactic acid and peptide-N compared with the control (*p* < 0.05). The acetic acid content was decreased (*p* < 0.05) in alfalfa silage only when prepared with GI19+GI51. No significant difference in the numbers of LAB, aerobic bacteria, or coliform bacteria were observed by LAB additions. Compared with LAB-additive treatments, upon further sucrose addition, the pH and counts of LAB and aerobic bacteria, as well as the contents of acetic acid and NH_3_-N, were decreased (*p* < 0.05), whereas yeast counts and the contents of DM and lactic acid were increased (*p* < 0.05). The coliform bacterial populations declined below the detection limit. Furthermore, the acetic acid content was lower (*p* < 0.05) in the GI19+GI51 silage than in the GI19 silage, whereas in the GI19+S silage, the yeast count and NH_3_-N content were lower than those in the GI19+GI51+S silage, but differences in the remnant indexes were not evident. Among all silage treatments, sucrose significantly affected all indexes of microbial populations, chemical compositions, fermentation characteristics, and NPN fractions, except for the contents of CP, NDF, ADF, NPN, and FAA-N, and LAB affected the yeast count and acetic acid content notably, while the interaction between sucrose and LAB affected the LAB count and contents of WSC, acetic acid, and NH_3_-N significantly.

## 4. Discussion

The abundance and presence of LAB on plants are related to plant species and environmental factors [3,26]. Owing to drought conditions and the large temperature difference between day and night in the native grassland of western Inner Mongolia, the survival of LAB is greatly challenged. Glucose, fructose, and sucrose are the preferred carbon sources for LAB, and are also the main carbon sources in phyllosphere, with average amounts of 12.5 µg/g of FM [27]. Most of the natural forages in this area have high lignin and low sugar contents, and due to limitations in wettability and/or diffusion of sugars across the leaf surface, the lack of available carbon sources may be the main factor for low number of LAB on the surface of some forages, which cannot be isolated by the plate method, especially those from Leguminosae, in which LAB strains were not detected on more than half of the species. The epiphytic LAB of forage plays a major role in silage fermentation, and legumes are difficult to ensile successfully on account of inadequate epiphytic LAB. Additionally, no LAB strains were isolated from fresh samples (four forage species belonging to three families) collected in Siziwang Banner, which reflected the fact that the distribution of epiphytic LAB was influenced by the geographic location [28]. The same epiphytic LAB species (*Ent. faecalis*) was observed on *Nitraria tangutorum* Bobr fresh samples growing in Urat back Banner and Urat Central Banner, respectively, and it was consistent with previous studies showing that the LAB inhabiting a plant’s surface may be related to the plant’s species [3]. In contrast, the LAB species dominating in *Nitraria tangutorum* Bobr silages were *Lati. graminis* and *Ped. acidilactici*, respectively. This result indicated that epiphytic LAB can be affected by the ensiling environment, and that some changes in the conditions of the ensiling process resulted in the succession of LAB species, strains, or both in silage [29].

Extensive 16S rDNA sequence analyses were performed to discriminate the LAB strains. According to molecular homology and classification, strains in groups A, B, C, D, G, H, I, J, K, N, P, Q, R, and T were identified unambiguously (Figure 3). However, these analyses were insufficient to classify the strains in groups E, F, L, M, O, and S at the species or subspecies level [18]. The use of housekeeping genes, such as *pheS* and *rpoA*, is emerging as an alternative to identify closely related species [30,31]. The *phe*S gene sequence analysis provided an interspecies gap, which normally exceeded 10%, and an interspecies variation of up to 3% divergence of members of the genus *Lactobacillus* [32]. The similarities of *phe*S gene between strains GI8, GI19, and the type strain of *Lact. plantarum* subsp. *plantarum* were 98.74~99.00%, while with respect to the type strains of other species or subspecies of *Lactobacillus* they were lower than 90.00%; thus, they were identified as *Lact. plantarum* subsp. *plantarum*. The *phe*S gene analysis also allowed the identification of the subspecies *Leuc. mesenteroides* subsp. *Jonggajibkimchii* [31]. The neighbour-joining trees derived from the 16S rDNA and *phe*S gene sequences revealed close relatedness between GI40 and *Leuc. mesenteroides* subsp. *Jonggajibkimchii* (Figure 3b and Figure 4a), with 100.00% and 99.20% of 16S rDNA and *phe*S gene sequence similarities, respectively, and the *phe*S gene sequence similarity was higher than the remaining subspecies of *Leuc. Mesenteroides* (≤98.93%); thus, it was identified as *Leuc. mesenteroides* subsp. *Jonggajibkimchii*. The *rpo*A gene has a higher discriminating power than the 16S rDNA sequence among closely related taxa of the *Enterococcus* genus. In the *Enterococcus* genus, strains of the same species have at least 99% *rpo*A gene sequence similarity, while different species have at maximum 97% *rpo*A gene sequence similarity [13]. Strain GI13 and the type strain of *Ent. casseliflavus* had 99.41% *phe*S gene sequence similarity, and for the other members of the genus *Enterococcus*, they were with a maximum of 94.00% *rpo*A gene sequence similarity, so GI13 was identified as *Ent. casseliflavus.* The *rpo*A gene proved discriminatory for the phylogenetic resolution of *Leuconostoc* spp. [33], and the strains GI62 and GI70 were identified with 99.74% similarity values as *Leu. mesenteroides* subsp. *suionicum*, which was higher than the type strains of other subspecies, with similarity values ranging from 97.77% to 98.35%.

Members of the *Enterococcus*, *Lactococcus*, *Lactobacillus*, *Pediococcus*, *Leuconostoc,* and *Weissella* genera are the most frequently detected LAB in plant tissues [26]; except for *Lactococcus*, all generas were detected in different forage plants in the native grassland of western Inner Mongolia and their spontaneous fermentation silages. Diverse LAB species were observed in this study, and 16 species were identified clearly. Strains of *Lact. paracasei*, *Liqu. sucicola*, *Ped. pentosaceus*, *W. paramesenteroides*, *W. cibaria*, *W. halotolerans*, and *Ent. mundtii* were 1.37% of the total isolates, respectively; strains of *Leuc. pseudomesenteroides*, *Ent. faecium* and *Ent. casseliflavus* accounted for 2.74% of total isolates, respectively; strains of *Lati. graminis* represented the 5.48% of total isolates; whereas strains of *Levi. brevis* and *Leuc. mesenteroides* accounted for 8.22% of all isolates, respectively. Strains of *Ped. acidilactici*, *Lact. plantarum*, and *Ent. faecalis* corresponded to 16.44%, 17.81%, and 26.03% of total isolates, respectively. *Enterococcus*, especially *Ent. faecalis* dominated on fresh forage plants, in agreement with Cai [11], while *Lact. plantarum* subsp. *plantarum* and *Ped. pentosaceus* were most commonly found in silage samples. In phenotypic characteristics clustering, almost all *Weissella* and *Leuconostoc* species were placed in the first cluster (Figure 2), which were also closely related in genetic clustering (Figure 3), and most remnant strains belonging to the same genus were grouped in a sub-cluster. These suggested LAB in one genus tended to possess similar phenotypic characteristics. Consistent with ecological specialisation, all isolates could grow at 5 °C, and well at 10 °C, except for strain GI16 in group R (Figure 2), indicating that their psychrophilic nature was selected by long-term evolution in cold weather [34]. Cold areas are always the main places to obtain LAB inoculants for promoting fermentation at low temperatures [34,35]. Moreover, the isolates, except those in group N and R, were all able to grow well or weakly in 6.5% NaCl, showing relatively higher osmotolerance, compared with previous studies [18,36]. Most forage plants in this arid region had high DM content. Their epiphytic LAB strains have lived long in arid climates, and form adaptations relevant to low water activity environments; some of them possess high osmotolerance to grow well in high DM silages prepared with forage plants. In high DM silages, the numbers of LAB growth are limited due to the shortage of available metabolic water that results in high pH [37]. Some *Ped. acidilactici* and *Ped. pentosaceus* strains show good growth at cold temperatures and have strong osmotolerance to thrive in conditions characterised by high DM content [8,35,38]. These properties might explain why *Ped. acidilactici* strains were most frequently isolated in natural forage plant silages of western Inner Mongolia. Furthermore, *W. halotolerans*, *Leuc. mesenteroides* subsp. *suionicum*, and *Leuc. mesenteroides* subsp. *jonggajibkimchii* were observed for the first time in fresh forage plant or/and silage, were all able to grow well in 6.5% NaCl, and always occur in fermented food containing salt [39,40]; especially for *W. halotolerans*, good growth could be demonstrated at 12% NaCl [41].

Because of the low LAB count and WSC content in raw alfalfa, an undesirable fermentation quality was obtained in spontaneous fermentation silage (Table 2). Thus, an appropriate inoculant should be introduced in ensiling to increase the success of alfalfa preservation. Among LAB, *Lactobacilli* are the most frequent isolates associated with silage because they play an essential role in promoting lactic acid fermentation, and are commonly used as inoculants for silage. Lactic acid-producing cocci, e.g., lactococci, leuconostocs, weissella, streptococci, pediococci, and enterococci, grow vigorously, start lactate fermentation, and inhibit undesirable microorganisms, which are suitable characteristics for the development of *Lactobacilli* [42]. Thus, two strains of *Lact. plantarum* subsp. *plantarum* (GI19) and *Ped. pentosaceus* (GI51) were screened for inoculation alone or in combination. The LAB inoculants effectively produced lactic acid and reduced the pH in alfalfa silages. In silages, propionibacteria and clostridia are able to convert sugars and lactic acid to propionic acid, butyric acid, or acetic acid, and the presence of these acids indicates large losses of DM and poor recovery of energy. However, the low pH from lactic acid stabilizes silage fermentation by inhibiting the growth of the propionibacteria and clostridia [37]. The contents of propionic acid were decreased compared with the control, and no butyric acid was detected. Thus, the LAB inoculants were positive for alfalfa silage quality. However, the pH only dropped to 4.76 to 4.81, consistent with a previous study [43], which was not enough to inhibit undesirable microorganisms such as aerobic bacteria and coliform bacteria, resulting in NH_3_-N content higher than 7.00% TN, and not meeting the silage with good quality [1]. However, the fermentation quality of alfalfa silages was further improved by LAB combined with sugar, compared with silages prepared with LAB alone, resulting in a lower pH and contents of acetic acid and NH_3_-N. This likely because the purpose of inoculating with LAB is to promote the efficient use of sugar, which can inhibit the growth of spoilage microorganisms and prevent undesirable processes such as acetic acid fermentation [44]. However, the lack of fermentation substrate in alfalfa silage weakens the fermentation efficiency of LAB and limits their function. Interestingly, the LAB inoculants combined with sucrose significantly increased the yeast count in alfalfa silage. Many yeasts can grow at pH 3.5, and some of them anaerobically ferment sugars to ethanol [45]. When sucrose was added, the plentiful of substrate promoted the production of lactic acid by LAB and decreased the content of acetic acid. Due to the accumulation of substrates and the reduction of inhibitors, beneficial conditions for some yeast were provided [46].

The paired LAB inoculant GI19+GI51 showed little effect on the improvement in silage quality compared with GI19 inoculation alone. Rooke and Kafilzadeh [47] found that the co-inoculation of *Pediococcus* with *Lact. plantarum* led to a silage dominated by homofermentative lactobacilli at day 16 and the growth of *Pediococcus* strains was suppressed. This observation demonstrated that GI19 had stronger competitiveness than GI51 under limited substrate conditions, because of its faster acid production rates and wider carbohydrate sources, which played a key role in ensiling alfalfa. While ensiling with sugar, the yeast count in the GI19+GI51+S silage was higher than that in the GI19+S silage. A plausible reason may be that under sufficient substrate, *Ped. pentosaceus* caused a faster reduction of silage pH to inhibit LAB earlier in the ensiling process, which resulted in more yeast development. Moreover, combined inoculation with GI19+GI51+S in silage resulted in a higher NH_3_-N content than that with GI19+S, likely due to a synergic effect in the presence of adequate substrate. *Ped. pentosaceus* inoculation did not affect the NH_3_-N concentration in silage [46], and its activity also weakened the inhibition of microbial deamination in silage compared with GI19+S. Thus, the combination of GI19 and GI51 did not translate to a positive synergy that could enhance silage fermentation. However, some combination of inoculants, including *Pediococcus* and *Lactobacillus* strains, reportedly show superior performance at low temperatures [34]. Considering the distinctive characteristics of the LAB isolated in this study, further research is needed to explore the effect of LAB inoculants on improving silage quality under harsh conditions, such as low temperature and/or low moisture.

## 5. Conclusions

In the native grassland of western Inner Mongolia, 73 strains of LAB were isolated from 30 forage species. These LAB isolates were identified and belonged to five genera and 16 species. *Ent. faecalis* and *Lact. plantarum* were the dominant species in fresh materials and silages, respectively. Affected by the environmental and plant conditions, most of the isolates showed good cryotolerance and osmotolerance. All additives in this study were positive for alfalfa silage, and co-inoculation of the *Lact. plantarum* subsp. *plantarum* strain GI19 with sugar was more effective for alfalfa ensiling. GI19+S is considered a suitable candidate for alfalfa ensiling in this area.

## Figures and Tables

**Table 1:** The morphological characteristics, fermentation types, and acidifications in MRS broth of representative strains isolated from forage plants and their silage samples.

Group	Number of Strains	Representative Strain	Cell Form	Fermentation Type	pH in MRS Broth ^1^		
					12 h	24 h	48 h
A	4	GI41	rod	Homo	4.65	4.30	4.23
B	1	GI4	rod	Homo	4.12	3.81	3.70
C	1	GI65	rod	Homo	4.20	3.98	3.95
D	6	GI56	rod	Hetero	5.50	5.09	4.80
E	1	GI8	rod	Homo	3.84	3.75	3.67
F	12	GI19	rod	Homo	3.98	3.88	3.80
G	1	GI51	cocci	Homo	4.10	4.05	3.90
H	12	GI50	cocci	Homo	4.59	4.22	4.13
I	1	GI46	cocci	Hetero	5.16	4.35	4.24
J	1	GI21	cocci	Hetero	4.66	4.29	4.17
K	1	GI38	cocci	Hetero	4.81	4.52	4.34
L	2	GI40	cocci	Hetero	4.82	4.19	4.20
M	1	GI62	cocci	Hetero	4.81	4.21	4.19
N	2	GI67	cocci	Hetero	5.03	4.36	4.32
O	3	GI70	cocci	Hetero	4.60	4.39	4.40
P	1	GI52	cocci	Homo	5.99	4.84	4.42
Q	2	GI22	cocci	Homo	5.27	4.35	4.32
R	1	GI16	cocci	Homo	5.24	4.91	4.32
S	1	GI13	cocci	Homo	5.04	4.91	4.50
T	19	GI30	cocci	Homo	5.09	4.54	4.43

^1^ Values are means of three samples.

**Table 2:** Microbial and chemical compositions of alfalfa prior to and after 60 d of ensiling prepared with lactic acid bacteria (LAB) strains and sucrose (S).

Item	Raw Alfalfa	Control	GI19	GI19+GI51	GI19+S	GI19+GI51+S	SEM	*p*-Value		
								A	L	A × L
Microbial populations (log_10_ cfu/g FM)										
LAB	3.30	7.14 ^a^	7.64 ^a^	7.41 ^a^	4.74 ^b^	5.19 ^b^	0.333	<0.001	0.512	0.067
Aerobic bacteria	5.10	8.22 ^a^	7.18 ^a^	7.52 ^a^	4.31 ^b^	5.12 ^b^	0.422	<0.001	0.059	0.403
Coliform bacteria	4.62	3.42 ^a^	3.14 ^ab^	2.72 ^ab^	ND	ND	0.133	0.047	0.380	0.380
Yeast	3.25	ND	ND	ND	4.74 ^b^	5.32 ^a^	0.362	<0.001	0.041	0.041
Mold	3.00	ND	ND	ND	ND	ND	--	--	--	--
Chemical compositions										
DM (%)	25.65	24.22 ^ab^	23.14 ^b^	23.13 ^b^	25.25 ^a^	25.40 ^a^	0.295	<0.001	0.812	0.803
CP (% DM)	17.87	16.06	17.29	17.41	17.20	17.46	0.212	0.973	0.693	0.878
NDF (% DM)	43.76	43.21	43.58	43.59	43.58	42.12	0.383	0.437	0.442	0.438
ADF (% DM)	32.68	32.21	32.29	32.04	32.63	31.03	0.316	0.647	0.229	0.365
WSC (% DM)	5.82	1.10 ^b^	1.08 ^b^	1.36 ^b^	2.38 ^a^	2.16 ^a^	0.150	<0.001	0.811	0.032
Fermentation characteristics										
PH	--	5.69 ^a^	4.81 ^b^	4.76 ^b^	3.91 ^c^	3.93 ^c^	0.181	<0.001	0.567	0.174
Lactic acid (% DM)	--	1.64 ^c^	3.62 ^b^	3.87 ^b^	7.82 ^a^	7.10 ^a^	0.650	<0.001	0.380	0.092
Acetic acid (% DM)	--	2.28 ^a^	2.22 ^a^	1.28 ^b^	0.31 ^c^	0.21 ^c^	0.252	<0.001	0.004	0.012
Propionic acid (% DM)	--	0.53 ^a^	0.04 ^b^	0.05 ^b^	0.01 ^b^	0.01 ^b^	0.062	0.015	0.658	0.658
Butyric acid (% DM)	--	0.77	ND	ND	ND	ND	--	--	--	--
Non-protein N fractions (NPN) (% TN)										
NPN	26.34	61.86	62.18	61.72	58.04	61.93	0.747	0.272	0.333	0.228
NH_3_-N	--	18.28 ^a^	10.49 ^b^	7.84 ^b^	1.22 ^d^	4.68 ^c^	1.500	<0.001	0.179	<0.001
FAA-N	--	29.00	27.00	27.01	24.29	25.95	0.637	0.104	0.442	0.442
Peptide-N		14.00 ^b^	24.69 ^a^	26.87 ^a^	32.52 ^a^	31.30 ^a^	1.959	0.039	0.851	0.512 ^1^

^1^ Means in the same row with different superscript letters are significantly different by Tukey’s multiple comparison method (*p* < 0.05). GI19, *Lactiplantibacillus plantarum* subsp. *plantarum*; GI51, *Pediococcus pentosaceus*. SEM, standard error of mean; A, effect of sucrose; L, effect of LAB; A × L, interaction between sucrose and LAB. DM, dry matter; CP, crude protein; NDF, neutral detergent fibre; ADF, acid detergent fibre; WSC, water-soluble carbohydrate; TN, total nitrogen; NH_3_-N, ammonia nitrogen; FAA-N, free amino acid N; ND, not detected.

## Data Availability

The original contributions presented in the study are included in the article/Supplementary Material, further inquiries can be directed to the corresponding author.

## Supplementary Materials

The following supporting information can be downloaded at: https://www.mdpi.com/article/10.3390/ani14101394/s1, Table S1: Geographical distribution of forage plants and plant-associated lactic acid bacteria in the native grassland of western Inner Mongolia.

## References

[1] McDonald P., Henderson N., Heron S. (1991). The Biochemistry of Silage.

[2] Muck R.E., Nadeau E., Mcallister T.A., Contrerasgovea F.E., Santos M.C., Kung L. (2018). Silage review: Recent advances and future uses of silage additives. J. Dairy Sci..

[3] Muck R. (2013). Recent advances in silage microbiology. Agric. Food Sci..

[4] Turner T.R., James E.K., Poole P.S. (2013). The plant microbiome. Genome Biol..

[5] Wagner M.R., Lundberg D.S., Del Rio T.G., Tringe S.G., Dangl J.L., Mitchell-Olds T. (2016). Host genotype and age shape the leaf and root microbiomes of a wild perennial plant. Nat. Commun..

[6] Lindow S.E., Brandl M.T. (2003). Microbiology of the Phyllosphere. Appl. Environ. Microbiol..

[7] Di Cagno R., Coda R., De Angelis M., Gobbetti M. (2013). Exploitation of vegetables and fruits through lactic acid fermentation. Food Microbiol..

[8] Kung Jr L. Silage Fermentation & Additives. https://www.researchgate.net/profile/Limin_Kung/publication/267421247_SILAGE_FERMENTATION_ADDITIVES/links/55ccc4ab08aecae56cc1c3b4/SILAGE-FERMENTATION-ADDITIVES.pdf?origin=publication_detail.

[9] Zhang Q., Yu Z., Wang X. (2015). Isolating and evaluating lactic acid bacteria strains with or without sucrose for effectiveness of silage fermentation. Grassl. Sci..

[10] Wang Y., Chen X., Wang C., He L., Zhou W., Yang F., Zhang Q. (2019). The bacterial community and fermentation quality of mulberry (*Morus alba*) leaf silage with or without *Lactobacillus casei* and sucrose. Bioresour. Technol..

[11] Cai Y. (1999). Identification and characterization of *Enterococcus* species isolated from forage crops and their influence on silage fermentation. J. Dairy Sci..

[12] Tohno M., Kobayashi H., Nomura M., Kitahara M., Ohkuma M., Uegaki R., Cai Y. (2012). Genotypic and phenotypic characterization of lactic acid bacteria isolated from Italian ryegrass silage. Anim. Sci. J..

[13] Naser S.M., Thompson F.L., Hoste B., Gevers D., Dawyndt P., Vancanneyt M., Swings J. (2005). Application of multilocus sequence analysis (MLSA) for rapid identification of *Enterococcus* species based on *rpoA* and *pheS* genes. Microbiology.

[14] Katoh K., Standley D.M. (2013). MAFFT Multiple Sequence Alignment Software Version 7: Improvements in Performance and Usability. Mol. Biol. Evol..

[15] Stamatakis A. (2014). RAxML version 8: A tool for phylogenetic analysis and post-analysis of large phylogenies. Bioinformatics.

[16] Letunic I., Bork P. (2016). Interactive tree of life (iTOL) v3: An online tool for the display and annotation of phylogenetic and other trees. Nucleic Acids Res..

[17] Kozaki M., Uchimura T., Okada S. (1992). Experimental Manual for Lactic Acid Bacteria.

[18] Li D., Wang Y., Zhang Y., Lin Y., Yang F. (2018). Evaluation of lactic acid bacteria isolated from alfalfa for silage fermentation. Grassl. Sci..

[19] Yang J., Tan H., Cai Y. (2016). Characteristics of lactic acid bacteria isolates and their effect on silage fermentation of fruit residues. J. Dairy Sci..

[20] Broderick G.A., Kang J.H. (1980). Automated simultaneous determination of ammonia and total amino acids in ruminal fluid and in vitro media1. J. Dairy Sci..

[21] Muck R.E. (1987). Dry matter level effects on alfalfa silage quality I. Nitrogen transformations. Trans. ASAE.

[22] Licitra G., Hernandez T.M., Soest P.J. (1996). Standardization of procedures for nitrogen fractionation of ruminant feeds. Anim. Feed Sci. Technol..

[23] AOAC (1990). Official Methods of Analysis.

[24] Murphy R.P. (1958). A method for the extraction of plant samples and the determination of total soluble carbohydrates. J. Sci. Food Agric..

[25] Van Soest P.V., Robertson J.B., Lewis B.A. (1991). Methods for dietary fiber, neutral detergent fiber, and nonstarch polysaccharides in relation to animal nutrition. J. Dairy Sci..

[26] Yu A.O., Leveau J.H., Marco M.L. (2020). Abundance, diversity and plant-specific adaptations of plant-associated lactic acid bacteria. Environ. Microbiol. Rep..

[27] Mercier J., Lindow S.E. (2000). Role of leaf surface sugars in colonization of plants by bacterial epiphytes. Appl. Environ. Microbiol..

[28] Fhoula I., Najjari A., Turki Y., Jaballah S., Ouzari H. (2013). Diversity and antimicrobial properties of lactic acid bacteria isolated from rhizosphere of olive trees and desert truffles of tunisia. BioMed Res. Int..

[29] Lin C., Bolsen K.K., Brent B.E., Fung D.Y.C. (1992). Epiphytic lactic acid bacteria succession during the pre-ensiling and ensiling periods of alfalfa and maize. J. Appl. Bacteriol..

[30] Liu D.D., Gu C.T. (2019). *Lactobacillus pingfangensissp*. nov., *Lactobacillus daoliensissp.* nov., *Lactobacillus nangangensissp*. nov., *Lactobacillus daowaiensissp*. nov., *Lactobacillus dongliensissp.* nov., *Lactobacillus songbeiensissp*. nov. and *Lactobacillus kaifaensissp*. nov., isolated from traditional Chinese pickle. Int. J. Syst. Evol. Microbiol..

[31] Sánchez-Juanes F., Teixeira-Martín V., González-Buitrago J.M., Velázquez E., Flores-Félix J.D. (2020). Identification of species and subspecies of lactic acid bacteria present in spanish cheeses type “torta” by MALDI-TOF MS and *pheS* gene analyses. Microorganisms.

[32] Naser S.M., Dawyndt P., Hoste B., Gevers D., Vandemeulebroecke K., Cleenwerck I., Vancanneyt M., Swings J. (2007). Identification of lactobacilli by *pheS* and *rpoA* gene sequence analyses. Int. J. Syst. Evol. Microbiol..

[33] Nel S., Davis S.B., Endo A., Dicks L.M. (2020). Phylogenetic analysis of *Leuconostoc* and *Lactobacillus* species isolated from sugarcane processing streams. MicrobiologyOpen.

[34] Wang S., Dong Z., Li J., Chen L., Shao T. (2018). Effects of storage temperature and combined microbial inoculants on fermentation end products and microbial populations of Italian ryegrass (*Lolium multiflorum* Lam.) silage. J. Appl. Microbiol..

[35] Xu D.M., Ke W.C., Zhang P., Li F.H., Guo X.S. (2019). Characteristics of *Pediococcus pentosaceus* Q6 isolated from *Elymus nutans* growing on the Tibetan Plateau and its application for silage preparation at low temperature. J. Appl. Microbiol..

[36] Pang H., Qin G., Tan Z., Li Z., Wang Y., Cai Y. (2011). Natural populations of lactic acid bacteria associated with silage fermentation as determined by phenotype, 16S ribosomal RNA and *recA* gene analysis. Syst. Appl. Microbiol..

[37] Kung L., Shaver R.D., Grant R.J., Schmidt R.J. (2018). Silage review: Interpretation of chemical, microbial, and organoleptic components of silages. J. Dairy Sci..

[38] Jin L., Chevaux E., McAllister T., Baah J., Drouin P., Wang Y. (2018). Impact of *Pediococcus pentosaceus* and *Pichia anomala* in combination with chitinase on the preservation of high-moisture alfalfa hay. Grass Forage Sci..

[39] Fusco V., Quero G.M., Cho G.S., Kabisch J., Meske D., Neve H., Bockelmann W., Franz C.M. (2015). The genus *Weissella*: Taxonomy, ecology and biotechnological potential. Front. Microbiol..

[40] Jeon H.H., Kim K.H., Chun B.H., Ryu B.H., Han N.S., Jeon C.O. (2017). A proposal of *Leuconostoc mesenteroides* subsp. *jonggajibkimchii* subsp. nov. and reclassification of *Leuconostoc mesenteroides* subsp. *suionicum* (Gu et al., 2012) as *Leuconostoc suionicum* sp. nov. based on complete genome sequences. Int. J. Syst. Evol. Microbiol..

[41] Kandler O., Schillinger U., Weiss N. (1983). *Lactobacillus halotolerans* sp. nov., nom. rev. and *Lactobacillus minor* sp. nov., nom. rev. Syst. Appl. Microbiol..

[42] Ennahar S., Cai Y., Fujita Y. (2003). Phylogenetic diversity of lactic acid bacteria associated with paddy rice silage as determined by 16S ribosomal DNA analysis. Appl. Environ. Microbiol..

[43] Blajman J.E., Vinderola G., Páez R.B., Signorini M.L. (2020). The role of homofermentative and heterofermentative lactic acid bacteria for alfalfa silage: A meta-analysis. J. Agric. Sci.

[44] Zheng M.L., Niu D.Z., Jiang D., Zuo S.S., Xu C.C. (2017). Dynamics of microbial community during ensiling direct-cut alfalfa with and without LAB inoculant and sugar. J. Appl. Microbiol..

[45] Muck R.E. (2010). Silage microbiology and its control through additives. Rev. Bras. Zootec..

[46] Oliveira A.S., Weinberg Z.G., Ogunade I.M., Cervantes A.A., Arriola K.G., Jiang Y., Kim D., Li X., Gonçalves M.C.M., Vyas D. (2017). Meta-analysis of effects of inoculation with homofermentative and facultative heterofermentative lactic acid bacteria on silage fermentation, aerobic stability, and the performance of dairy cows. J. Dairy Sci..

[47] Rooke J.A., Kafilzadeh F. (1994). The effect upon fermentation and nutritive value of silages produced after treatment by three different inoculants of lactic acid bacteria applied alone or in combination. Grass Forage Sci..

