# Supplementary material for: Genotypic and Phenotypic Characteristics of Lactic Acid Bacteria Associated with Forage Plants in the Native Grassland of Western Inner Mongolia and Their Application for Alfalfa Silage Fermentation
Source: Animals (Basel). 2024 May 7;14(10):1394. doi: 10.3390/ani14101394 (PMC11117391; doi:10.3390/ani14101394)
Supplement: Supplementary file 1 [file animals-14-01394-s001.zip › animals-2931093-supplementary.pdf]

Table S1 Geographical distribution of forage plants and plant-associated lactic acid bacteria in the native grassland of western Inner Mongolia

Plant family	Natural forages	Geographic coordinates	Materials	
			Fresh material	60 d silage
Leguminosae	*Caragana stenophylla* Pojark	Urat Back Banner, Bayannur (41°40′N, 107°04′E)	GI2	GI3, GI4
	Ammopiptanthus mongolicus (Maxim.) Cheng f.	Urat Back Banner, Bayannur (41°14′N, 103°37′E)		GI5
	Caragana microphylla Lam.	Siziwang Banner, Wulanchabu (42°36′N, 111°50′E)		GI7, GI8
	Caragana intermedia Kuang et H.C. Fu	Siziwang Banner, Wulanchabu (41°71′N, 111°63′E)		GI9
	Alhagi maurorum Medic. var.sparsifolium (Shap.) Yakovl.	Ejin Banner, Alashan (40°04′N, 101°06′E)		GI10, GI11
	Hedysarum scoparium Fisch. Et Mey.	Hanggin Banner, Ordos (40°58′N, 108°74′E)		GI12
	Hedysarum laevc Maxim	Hanggin Banner, Ordos (40°00′N, 107°89′E)	GI13	GI14
	Ammopiptanthus mongolicus (Maxim.) Cheng f.	Alxa Left Banner, Alashan (39°62′N, 105°10′E)		GI15
	Sophora alopecurides L.	Ejin Banner, Alashan (41°93′N, 101°03′E)	GI16, GI17	GI18, GI19
Chenopodiaceae	*Salsola passerina* Bunge	Urat Back Banner, Bayannur (41°16′N, 106°49′E)	GI20	
	Anabasis brevifolia C.A. Mey.	Urat Back Banner, Bayannur (41°72′N, 106°37′E)		GI21
	Zygophyllum pterocarpum	Urat Back Banner, Bayannur (41°16′N, 106°49′E)		GI22
	Eurotiac eratoides (L.) Mey.	Urat Back Banner, Bayannur (41°40′N, 107°04′E)		GI23, GI24
	Haloxylon ammodendron (C. A. Mey.) Bunge	Urat Back Banner, Bayannur (41°40′N, 107°04′E)	GI25	GI26, GI27
	Nitraria tangutorum Bobr	Urat Back Banner, Bayannur (41°14′N, 103°37′E)	GI28	GI29
	Nitraria tangutorum Bobr	Urat Back Banner, Bayannur (41°16′N, 106°49′E)	GI39	

Table S1 Geographical distribution of forage plants and plant-associated lactic acid bacteria in the native grassland of western Inner Mongolia (continued table)

Plant family	Natural forages	Geographic coordinates	Materials	
			Fresh material	60 d silage
Chenopodiaceae	*Nitraria tangutorum* Bobr	Urat Central Banner, Bayannur (42°36′N, 107°77′E)	GI30	GI31
	Zygophyllum pterocarpum	Urat Central Banner, Bayannur (42°36′N, 107°77′E)	GI32	GI33
	Kalidium gracile Fenzl.	Urat Central Banner, Bayannur (42°36′N, 107°78′E)	GI34	GI35, GI36
	Anabasis brevifolia C.A. Mey.	Urat Central Banner, Bayannur (42°36′N, 107°78′E)	GI37	GI38
	Haloxylon ammodendron (C. A. Mey.) Bunge	Alxa Left Banner, Alashan (39°30′N, 105°70′E)		GI40, GI41
	Atriplex patens (Litv.) lljin.	Alxa Right Banner, Alashan (38°99′N, 101°77′E)		GI42, GI43
Asteraceae	*Ajania achilleoides* (Turcz.) Poljak. Et Grub	Urat Back Banner, Bayannur (41°40′N, 107°04′E)		GI44
	Echinops gmelinii Turcz.	Urat Back Banner, Bayannur (42°36′N, 107°77′E)	GI45	GI46
	Artemisia sieversiana Ehrhart ex Willd.	Darhan Muminggan Joint Banner, Baotou (42°61′N, 110°28′E)	GI47, GI48	GI49, GI50
	Artemisia frigida Willd.	Siziwang Banner, Wulanchabu (42°39′N, 111°37′°E)		GI51
	Karelinia caspia (Pall.) Less.	Ejin Banner, Alashan (40°04′N, 101°06′E)	GI52	GI53
	Asterothamnus centrali-asiaticus Novopokr.	Alxa Left Banner, Alashan (39°04′N, 105°99′E)	GI54	GI55
	Heteropappus altaicus (Willd.) Novopokr.	Alxa Left Banner, Alashan (39°04′N, 105°99′E)		GI56, GI57
Zygophyllaceae	*Peganum harmala* L.	Urat Back Banner, Bayannur (41°14′N, 103°37′E)	GI58	GI59
Lamiaceae	*Lagochilus ilicifolius* Bunge	Urat Back Banner, Bayannur (41°40′N, 107°04′E)	GI60	GI61, GI62
Cruciferae	*Lepidium latifolium* L.	Urat Central Banner, Bayannur (42°36′N, 107°77′E)	GI63	GI64, GI65

Table S1 Geographical distribution of forage plants and plant-associated lactic acid bacteria in the native grassland of western Inner Mongolia (continued table)

Plant family	Natural forages	Geographic coordinates	Materials	
			Fresh material	60 d silage
Liliaceae	*Alliun polyrhizum* Turcz. ex Regel (florescence)	Siziwang Banner, Wulanchabu (42°36′N, 111°50′E)		GI66
	Alliun polyrhizum Turcz. ex Regel (vegetative period)	Siziwang Banner, Wulanchabu (42°39′N, 111°37′E)		GI67, GI68
Iridaceae	*Iris lactea Pall. Var. chinensis* (Fisch.) Koidz.	Darhan Muminggan Joint Banner, Baotou (42°61′N, 110°28′E)		GI69, GI70
Rosaceae	*Prunus mongolica* Maxim.	Dengkou, Bayannur (40°58′N, 106°32′E)	GI71, GI72	GI73
Caryophyllaceae	Gymnocarpos przewalskii Maxim.	Alxa Right Banner, Alashan (38°99′N, 101°77′E)	GI74	GI1
